# Microglial Dysregulation in OCD, Tourette Syndrome, and PANDAS

**DOI:** 10.1155/2016/8606057

**Published:** 2016-12-07

**Authors:** Luciana Frick, Christopher Pittenger

**Affiliations:** ^1^Department of Psychiatry, Yale University, New Haven, CT, USA; ^2^34 Park Street, 3rd floor, W306, New Haven, CT 06519, USA; ^3^Department of Psychology, Yale University, New Haven, CT, USA; ^4^Department of Interdepartmental Neuroscience Program, Yale University, New Haven, CT, USA; ^5^Department of Child Study Center, Yale University, New Haven, CT, USA

## Abstract

There is accumulating evidence that immune dysregulation contributes to the pathophysiology of obsessive-compulsive disorder (OCD), Tourette syndrome, and Pediatric Autoimmune Neuropsychiatric Disorders Associated with Streptococcal Infections (PANDAS). The mechanistic details of this pathophysiology, however, remain unclear. Here we focus on one particular component of the immune system: microglia, the brain's resident immune cells. The role of microglia in neurodegenerative diseases has been understood in terms of classic, inflammatory activation, which may be both a consequence and a cause of neuronal damage. In OCD and Tourette syndrome, which are not characterized by frank neural degeneration, the potential role of microglial dysregulation is much less clear. Here we review the evidence for a neuroinflammatory etiology and microglial dysregulation in OCD, Tourette syndrome, and PANDAS. We also explore new hypotheses as to the potential contributions of microglial abnormalities to pathophysiology, beyond neuroinflammation, including failures in neuroprotection, lack of support for neuronal survival, and abnormalities in synaptic pruning. Recent advances in neuroimaging and animal model work are creating new opportunities to elucidate these issues.

## 1. Introduction

Neuroimmune interactions are increasingly appreciated as both an important regulator of normal brain development and function and a potential contributor to the pathophysiology of a range of neuropsychiatric illnesses. In particular, there is accumulating evidence that immune dysregulation can contribute to obsessive-compulsive disorder (OCD) and Tourette syndrome, at least in a subset of cases [1, 2]. The mechanistic details of this pathophysiology, however, remain unclear.

OCD is characterized by unreasonable or excessive thoughts and fears (obsessions) and/or repetitive behaviors (compulsions) [3]. Tourette syndrome, which is frequently comorbid with OCD, is characterized by tics: repetitive, stereotyped, involuntary movements and vocalizations [4, 5]. Both OCD and Tourette syndrome are accompanied by pathological changes in the corticobasal ganglia circuitry, especially in the striatum [6, 7].

A role for dysregulated immune function is particularly clear in the syndrome known as Pediatric Autoimmune Neuropsychiatric Disorders Associated with Streptococcal Infections, or PANDAS [8]. PANDAS is characterized by the sudden onset of OCD and/or tic symptoms in childhood, following a streptococcal infection [9, 10]. The symptoms are usually dramatic and can include motor and vocal tics, obsessions, and compulsions. It has been hypothesized that PANDAS arises from the development of brain-reactive autoantibodies after infection with Group A* Streptococcus* [10].

Here we review the evidence for an immunological etiology for OCD, Tourette syndrome, and related conditions. We focus on one particular component of the immune system: microglia, the brain-resident immune cells. These enigmatic cells have recently emerged as potential key players in the pathophysiology of neuropsychiatric disorders [11].

### 1.1. New Insights into Microglia Function

Microglia are the resident immune cells in the brain. Their activation in neurological disease has classically been associated with inflammation, neuronal damage, and neurodegeneration. However, over the past decade, novel roles for microglia in brain development, homeostasis, and plasticity have emerged [12]. A groundbreaking study demonstrated that microglia can engulf synapsis during normal postnatal development in mice [13]. Synaptic pruning by microglia is necessary for the formation of brain circuitry and normal connectivity [14]. Disruption of neuron-microglia interactions, through disruption of the fractalkine/fractalkine receptor signaling pathway, results in a range of neural and behavioral abnormalities [15]. Microglia cells are also necessary for adult neurogenesis [16] and provide support for neuronal survival [17].

The role of microglia in neurodegenerative conditions has been very well studied. More recently, in keeping with our growing understanding of their roles in modulation of normal brain function, research has focused on neuropsychiatric conditions that are not characterized by frank neuronal death [11]. Microglial contributions to pathophysiology in these disorders may be subtle and may relate to their noninflammatory functions. As new functions of microglia in normal brain development and function are discovered, and disruption of these functions in disease is characterized, new therapeutic strategies will emerge.

### 1.2. Microglial Abnormalities in Animal Models of Repetitive Behavioral Pathology

A number of animal models have been described in recent years in which the primary behavioral pathology is a maladaptive excess of repetitive behaviors, most commonly grooming [18]. These have often been interpreted as modeling OCD [19–21], but repetitive grooming has also been described in models of Tourette syndrome [22], autism [23], Rett syndrome [24], trichotillomania, and other conditions [18]. We first review several models in which the precise disease correlate is less firmly established but the association between microglial abnormalities in the corticostriatal circuitry is particularly striking. Animal studies with clearer etiopathogenic links to particular diseases are reviewed subsequently, together with the relevant clinical literature.

An early study reported that knockout of the* Hoxb8* gene produces compulsive grooming, progressing to hair removal and ultimately to skin lesions [19].* Hoxb8* expression in the brain is restricted to microglia (identified in these experiments by their expression of the cell-surface marker CD11b^+^). Strikingly, abnormal behavior in* Hoxb8* knockouts (KOs) is rescued by transplantation of normal bone marrow, which repopulates the brain with wild-type microglia. Conversely, excessive grooming is produced by transplantation of bone marrow from* Hoxb8* KO mice into wild-type animals [25].

Interestingly, not all CD11b^+^ cells in the brain express* Hoxb8*, which suggests that* Hoxb8* ^+^ cells might be a subpopulation of microglia, constituting approximately 40% of total microglial cells. However, the total number of microglia cells in the brain of* Hoxb8* mutant mice is reduced by only 15%. This finding raises two hypotheses. First, the Hoxb8^+^ subpopulation may be necessary for maintaining normal brain function, and loss of these cells in the KO animals may produce pathological grooming. Second, the population of* Hoxb8*-negative microglia may expand in the knockout animals, which may lead to functional abnormalities.

In progranulin deficient (*Grn* ^−/−^) mice, microglial activation leads to both excessive grooming and neurodegeneration [26]. This led to the suggestion that their repetitive behavior could be more related to classic inflammatory microglial activation and neuronal damage, rather than the loss of a neuroprotective or neuromodulatory function. Excessive grooming is associated with hyperexcitability of thalamocortical circuits in these mice. Notably, autosomal dominant mutations in the human* GRN* gene contribute to a common form of familial frontotemporal lobar degeneration [27, 28]. No association of* GRN* mutations with OCD, Tourette syndrome, or related conditions has been described.

CD11b^+^ cells in the brain are all of monocyte lineage, but they can be either resident brain microglia or peripherally derived monocytes. Although both resident microglia and peripherally derived monocytes express the CX3CR1 [29, 30], only the latter express CCR2, providing a powerful marker to discriminate between resident microglia and infiltrating monocyte in the brain. CX3CR1^+^ microglia are widely distributed through the brain parenchyma, while CCR2^+^ monocytes are rarely seen in the healthy brain [29]. Interestingly, microglia and monocytes play differential roles in neurodegeneration and brain injury [31–33]. Distinguishing between these two populations may therefore prove to be quite important in understanding how microglial abnormalities can lead to repetitive behavioral pathology.

In* Hoxb8 *KO mice, the fact that abnormal behavior is rescued by transplanting in wild-type bone marrow [25] indicates that Hoxb8^+^ cells derived from circulating monocytes can enter the brain and have behavioral effects. This is consistent with evidence indicating that Hoxb8 regulates monocyte/macrophage differentiation from hematopoietic precursors [34–36]. On the other hand, another recent paper showed that mice lacking CX3CR1, which is expressed by brain-resident microglia, show excessive grooming, along with other behavioral abnormalities that were interpreted as representing an autism-spectrum disorder-like phenotype [15].

### 1.3. Microglia Activation in Tourette Syndrome

Considerable evidence suggests that immune dysregulation may contribute to the pathophysiology Tourette syndrome [37, 38]. Evidence of microglial abnormalities, specifically, is much more limited. However, two recent studies, using different methodologies, have suggested abnormalities in microglial activation in patients with Tourette syndrome. Both studies focused on the basal ganglia.

In a recent postmortem analysis of brains from Tourette syndrome cases, Lennington et al. [39] described increased number of CD45^+^ microglial cells in the striatum. These cells had morphological changes consistent with neurotoxic activation, concomitant with enriched expression of inflammatory genes [39, 40]. Importantly, the brain samples were obtained from refractory adult patients; no comparable postmortem data exist for more typical pediatric and/or fluctuating disease.

A second recent study used in vivo positron emission tomography (PET) imaging with (^11^)C-[R]-PK11195 (PK), a ligand that binds to the transporter protein (TSPO), which is expressed by activated microglia. Increased PK binding, indicative of inflammatory microglial activation, was observed in the caudate nuclei bilaterally in children with Tourette syndrome [41]. An important caveat is that children with Tourette syndrome were compared to adult healthy controls (mean age 11.4 years versus mean age 28.7 years). Nevertheless, as the first study to image microglial activation in vivo in Tourette syndrome, this is an important advance.

Work in animal models of tic disorders may help to elucidate the role of microglia in their pathophysiology. Until recently, there have been no animal models of Tourette syndrome with clear links to its etiopathophysiology in which to do such work [42]. Fortunately, this situation is changing.

Tourette syndrome, like most neuropsychiatric conditions, is substantially genetic. Recent work has identified a hypomorphic mutation in* L-histidine decarboxylase *(*Hdc*), which encodes the rate-limiting enzyme in the biosynthesis of histamine, as a rare but high-penetrance genetic cause of Tourette syndrome [43]. Knockout of the* Hdc *gene, which recapitulates this molecular abnormality, thus produces an animal model with strong etiologic validity. These mice exhibit behavioral and neurochemical abnormalities seen in patients with Tourette syndrome, further confirming the validity of the model [44, 45].

We have recently described intriguing abnormalities in microglia activation in this model. We found that microglia cells are not activated in basal conditions in the striatum in* Hdc*-KO mice; rather, microglia from this mice exhibit reduced arborization and normal expression of inflammatory markers [46]. A similar effect is seen when neuronally derived histamine is specifically disrupted, through targeted virus-mediated ablation of histaminergic neurons in the posterior hypothalamus [46]. The total number of microglia is unchanged in KO animals, but the number of microglia expressing Insulin-like Growth Factor 1 (IGF-1) is reduced. IGF-1 expressing microglia are necessary for neuronal survival and promote neurogenesis in nonpathological conditions [16, 17]; a specific reduction of these cells suggests impairment in these functions.

Inflammatory challenge with bacterial lipopolysaccharide (LPS) dramatically changed this pattern: microglia activation in the striatum was enhanced in* Hdc-KO* mice, compared with wild-type controls. This was accompanied by enhanced induction of the proinflammatory cytokines IL-1*β* and TNF-*α*. Taken together, these findings suggest that in this mouse model of Tourette syndrome there is a deficit in microglia-mediated neuroprotection, accompanied by overreactivity to environmental challenge. Such complex mechanisms cannot be appreciated in human studies and reinforce the importance of work in animal models to clarify the mechanisms of microglial dysregulation in neuropsychiatric disease.

The normal number of microglia in the* Hdc*-KO model and their reduced arborization at baseline [46] contrast with the more numerous and activated microglia seen postmortem [39]. It is possible that the activation of microglia observed in patients with Tourette syndrome emerges only after challenge by environmental factors, such as infection, or over the course of aging. Our studies in mice [44, 46] are performed in a pathogen-free environment, in young adult mice. After LPS challenge, microglia activation in the animal model much more closely resembles that seen postmortem in humans. Regardless of this consideration, all the studies point to activation of microglia in the basal ganglia in Tourette syndrome [39, 41, 46], irrespective of the complexity of the underlying mechanisms.

### 1.4. Microglial Abnormalities in Obsessive-Compulsive Disorder

OCD is highly comorbid with Tourette syndrome. Some evidence suggests an immunological contribution to its pathogenesis [47]. In aggregate, however, this evidence is much weaker than in the case of Tourette syndrome, except in the case of PANDAS, to which we return below. More particularly, the role of microglia cells in OCD has not been clearly elucidated. The* Hoxb8 *knockout mouse, described above [19, 25], has been described as a mouse model of OCD and may thus implicate microglial dysregulation in the disorder; however, clear clinical data linking abnormalities in the* Hoxb8* gene, or the consequences of its disruption, to OCD has yet to emerge. To date, there are no imaging or postmortem studies describing microglia abnormalities in patients with OCD.

### 1.5. Microglia in PANDAS/PANS

Obsessive-compulsive disorder (OCD) and Tourette syndrome often strike in childhood. In a subset of cases, acute onset temporally coincides with a bout of infectious disease, particularly with* Streptococcus*; this clinical syndrome is known as PANDAS, or more generally Pediatric Acute-onset Neuropsychiatric Syndrome (PANS). By analogy with the better-understood pathophysiology of rheumatic fever and Sydenham's chorea, OCD and Tourette syndrome symptoms in these cases have been hypothesized to arise from the development of autoantibodies that cross-react with proteins normally expressed in the brain; this mechanism is known as molecular mimicry. While many details of PANDAS as a clinical entity remain unclear, and some are controversial, the association of immune dysregulation with OCD and Tourette syndrome symptoms in this subset of pediatric patients is increasingly clear [10].

The TSPO/PK PET imaging study of microglial activation described above examined both noninfectious Tourette syndrome and PANDAS [41]. Children with PANDAS had increased PK binding in the striatum with respect to adult healthy controls; this corresponds with increased striatal volumes previously described during acute illness in PANDAS patients [48, 49]. Inflammation was higher and more broadly spread through the bilateral caudate and lentiform nucleus in PANDAS than in non-PANDAS Tourette syndrome. Importantly, this comparison with age-matched patients avoids the interpretive difficulties created by comparison to an adult healthy control group; the observed differences support the notion that PANDAS is etiologically distinct from non-PANDAS Tourette syndrome.

A recent study in mice examined the effects of intranasal Group A streptococcal (GAS) infection and may begin to shed light on the role of microglia in PANDAS. Repeated intranasal GAS inoculations result in increasing the number of CD68^+^/Iba1^+^ activated microglia in the glomerular layer of the olfactory bulb [50]. Abnormal synaptic pruning, probably mediated by microglia, was also observed (see below). The majority of activated microglia were found in close proximity to CD4^+^ T cells, suggesting that GAS antigens could be presented to Th17 cells by local microglia.

## 2. Possible Mechanisms

The role of microglia in neurodegenerative diseases has been understood in terms of classic, inflammatory activation, which may be both a consequence and a cause of neuronal damage. In OCD and Tourette syndrome, which are not characterized by frank neural degeneration, the nature of any contribution of microglial dysregulation to pathophysiology is much less clear. We explore a few possible mechanisms here.

### 2.1. Neuroprotection versus Neurodegeneration

It is increasingly clear that microglia function in both neuroprotection and neurodegeneration. We have described complex abnormalities of microglia in a mouse model of Tourette syndrome [46]. Naïve* Hdc-KO* mice display morphological abnormalities in striatal microglia that suggest quiescence, normal expression of inflammatory markers, but a reduced number of IGF-1 expressing microglia.

**Figure 1:**
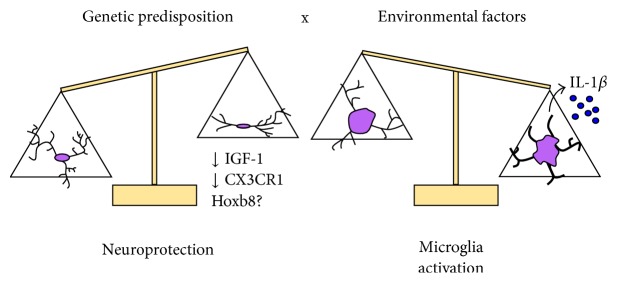
Gene X environment interaction in the pathophysiology of abnormal repetitive behaviors. Under normal conditions, IGF-1 expressing microglia provide neuroprotection, communicating with neurons via the CX3CL1/CX3CR1 signaling pathway. Failure of these interactions may lead to impaired neuroprotective support. In this context, in the face of an environmental stimulus, like an immunological challenge, dysregulated inflammatory microglial activation may lead to the induction of inflammatory cytokines such as IL-1*β* and to neuronal damage.

IGF-1 expression by microglia is induced by T_H_2 cytokines such as IL-4, which induce a neuroprotective phenotype, at least in organotypic hippocampal cultures [51]. In vivo, IGF-1 expressing microglia support cortical neurons during development and promote neurogenesis in the adulthood [16, 17, 52]. IGF-1 positive microglia protect the brain in neurodegenerative conditions [53, 54]. Thus, deficiency of IGF-1^+^ microglia in this animal model of Tourette syndrome might lead to impaired neuroprotection and, consequently, to enhanced susceptibility to neuroinflammation after an environmental challenge (Figure 1). Consistent with this, LPS challenge triggers an exaggerated response in* Hdc*-KO mice, both at the level of morphological activation and the production of the inflammatory cytokine IL-1*β* (Figure 1).

More generally, these results suggest that, in some neuropsychiatric disorders in which no marked neurodegeneration occurs, microglial dysregulation may constitute a failure of neuroprotective functions, which may create a vulnerability to neuroinflammation. This mechanism may explain observations in other animal studies, in which loss of microglia-specific genes triggers abnormal grooming behavior [15, 25]. For example, CX3CR1 is a key molecule for neuron-glia communication and has a role in neuroprotection [55–57], which supports the interpretation that knocking it out disrupts neuroprotective or regulatory functions of microglia (Figure 1).

### 2.2. Microglia and Neuronal Loss in Tourette Syndrome

**Figure 2:**
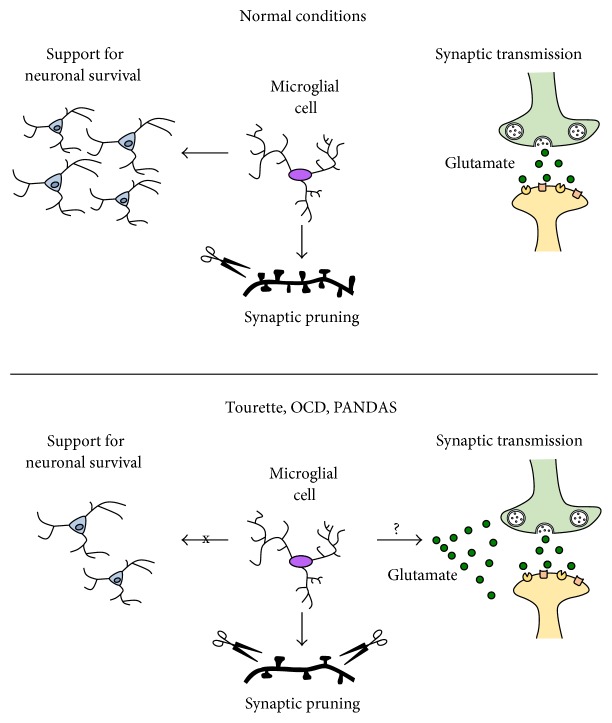
Possible mechanisms of abnormal microglial functions in OCD, Tourette syndrome, and PANDAS. Microglial cells support neuronal survival, and deficiencies in IGF-1 expressing microglia might lead to interneuronal loss (as observed in Tourette syndrome) or to abnormalities in synaptic pruning (as seen in animal models of GAS infection and excessive grooming). Microglial dysregulation may also lead to alterations in glutamate homeostasis, a phenomenon that occurs in OCD.

In postmortem Tourette's samples, loss of certain types of interneurons has been reported in the basal ganglia [39, 58, 59]. We have found reduced IGF-1 expressing microglia in the striatum in the* Hdc*-KO model of Tourette syndrome pathophysiology [46]. These IGF-1^+^ microglial cells are required for neuronal support during postnatal development, at least in the cortex [16, 17, 52]. Although these phenomena have not been linked, it is plausible that impaired IGF-1^+^ microglial function in the maintenance of striatal neurons might be causative of neuronal loss observed in Tourette syndrome (Figure 2).* Hdc*-KO mice have not been reported to develop spontaneous reduction of striatal interneurons; however, this might happen in aging mice or young mice subjected to immune challenge. In fact, Hdc-KO microglia are more susceptible to LPS-induced activation than wild-type microglia [46]. Further research is necessary to address this hypothesis.

### 2.3. Synaptic Pruning by Microglia

Recent studies have described a key role for microglia in synaptic pruning during development, with long lasting consequences in adulthood [13, 14]. CX3CR1 knockout mice exhibit deficient synaptic pruning, excessive grooming, and social deficits [15]. Whether this function is altered in OCD, Tourette syndrome, or PANDAS remains unclear. Progranulin KO mice, on the other hand, have abnormal microglial activation and increased synaptic pruning, which results in elimination of inhibitory synapses in the ventral thalamus (Figure 2), hyperexcitability in the thalamocortical circuits, and repetitive behavioral pathology.

As mentioned above, there is limited but promising evidence from animal studies that synaptic pruning might be altered in PANDAS. Using an animal model of intranasal GAS infection, Dileepan and coworkers observed microglial activation and loss of vGluT2, a marker of excitatory synapses [50]. Levels of the inhibitory marker GAD67 were normal. These results raise the possibility that synaptic pruning of excitatory connections may be increased in PANDAS (Figure 2).

Microglia are highly dynamic [60]. Their contact instances with neuronal synapses are reduced in frequency by reductions in neuronal activity [61, 62]. In the aging retina, for example, microglia become less dynamic and consequently morphologically less ramified. This may lead to impairments in their surveillance ability [63, 64]. In the* Hdc-KO* mouse, striatal microglia present similar morphological abnormalities [46]. It is possible that microglial abnormalities in this animal model lead to alterations of synaptic pruning. Preliminary results suggest that* Hdc*-KO animals may have CX3CR1 deficiencies (Frick et al., unpublished), which could be associated with abnormal synaptic pruning [15]. CX3CR1 deficiency produces altered microglial morphology, similar to what is seen in* Hdc*-KO mice [65]. Investigation of synaptic pruning in* Hdc*-KO mice, and other mouse models of Tourette syndrome, is warranted.

### 2.4. Glutamate, OCD, and Microglia

Increasing evidence suggests a pathophysiologically important dysregulation of glutamatergic signaling in patients with OCD [66]. Interactions between glutamate dysregulation and microglial abnormalities have not been investigated in this context. Such interactions have, however, been described in Rett syndrome, an autism-spectrum disorder characterized by mutation of the methyl-CpG-binding protein-2 gene (*MECP2*). MECP-null microglia release dramatically higher levels of glutamate, and microglia-derived glutamate has neurotoxic effects on dendrites and synapses [67]. It is plausible that microglia-derived glutamate might similarly contribute to the pathophysiology of OCD (Figure 2). Whether microglial abnormalities in OCD and Tourette syndrome, or in any of the animal models described above, are associated with glutamate dysregulation is an important area for future study.

## 3. Conclusions and Future Directions

While evidence for microglial dysregulation in OCD, Tourette syndrome, and PANDAS is growing, much remains unclear. The case for microglial dysregulation is strongest in the case of Tourette syndrome; recent postmortem and PET imaging studies have produced convergent evidence for increased microglial activation in the striatum in patients [40–42]. This is complemented by our studies in the* Hdc*-KO mouse model of Tourette syndrome [47]. We have proposed that these mice capture a pathophysiologically important gene X environment interaction, in which deficiency in microglia-mediated neuroprotection produces a susceptibility to inflammatory changes upon environmental challenge. This hypothesis needs to be explored in other well-validated models and in clinical contexts to test its generality.

Mechanisms by which microglial abnormalities contribute to disease are likely to be shared across distinct etiologies and traditional diagnoses. For instance, abnormal synaptic pruning was observed both in animals inoculated with GAS (which may capture key elements of the pathophysiology of PANDAS) and in mice that develop excessive grooming after inactivation of the progranulin gene. In both cases, increased synaptic pruning cooccurs with microglia activation. In CX3CR1 knockout mice, deficient synaptic pruning accompanies alterations in neuron-microglial communication. This is accompanied by social deficits, which has been interpreted as an autism-like phenotype. Results from these models must be interpreted cautiously, as neither progranulin nor CX3CR1 has been clearly associated with any of these conditions in humans. Abnormal synaptic pruning by microglia in the more pathophysiologically grounded* Hdc*-KO model of Tourette syndrome is warranted.

These studies in animal models are intriguing and allow detailed mechanistic analysis, but their relevance for the understanding of clinical disease remains to be firmly established. Information about microglial abnormalities in patients with OCD, Tourette syndrome, and PANDAS remains very limited. This is a recently opened frontier in our understanding of the pathophysiology of these disorders. It is, however, one of great promise, which may lead to the identification of novel therapeutic targets.
